# A Critical Period for Prefrontal Network Configurations Underlying Psychiatric Disorders and Addiction

**DOI:** 10.3389/fnbeh.2020.00051

**Published:** 2020-04-07

**Authors:** Ramon Guirado, Marta Perez-Rando, Antonio Ferragud, Nicolas Gutierrez-Castellanos, Juzoh Umemori, Hector Carceller, Juan Nacher, Esther Castillo-Gómez

**Affiliations:** ^1^Neurobiology Unit, Department of Cell Biology, Interdisciplinary Research Structure for Biotechnology and Biomedicine (BIOTECMED), Universitat de Valencia, Valencia, Spain; ^2^Neuroscience Center, University of Helsinki, Helsinki, Finland; ^3^Spanish National Network for Research in Mental Health, Centro de Investigación Biomédica en Red de Salud Mental (CIBERSAM), Madrid, Spain; ^4^Dirección General de Universidades, Gobierno de Aragón, Zaragoza, Spain; ^5^MassGeneral Institute for Neurodegenerative Disease, Massachusetts General Hospital and Harvard Medical School, Boston, MA, United States; ^6^Department of Psychology, Cambridge University, Cambridge, United Kingdom; ^7^Champalimaud Research Program, Lisbon, Portugal; ^8^Fundación Investigación Hospital Clínico de Valencia, INCLIVA, Valencia, Spain; ^9^Department of Medicine, School of Medical Sciences, Universitat Jaume I, Valencia, Spain

**Keywords:** prefrontal networks, decision-making, critical period, ventral hippocampus, basolateral amygdala

## Abstract

The medial prefrontal cortex (mPFC) has been classically defined as the brain region responsible for higher cognitive functions, including the decision-making process. Ample information has been gathered during the last 40 years in an attempt to understand how it works. We now know extensively about the connectivity of this region and its relationship with neuromodulatory ascending projection areas, such as the dorsal raphe nucleus (DRN) or the ventral tegmental area (VTA). Both areas are well-known regulators of the reward-based decision-making process and hence likely to be involved in processes like evidence integration, impulsivity or addiction biology, but also in helping us to predict the valence of our future actions: i.e., what is “good” and what is “bad.” Here we propose a hypothesis of a critical period, during which the inputs of the mPFC compete for target innervation, establishing specific prefrontal network configurations in the adult brain. We discuss how these different prefrontal configurations are linked to brain diseases such as addiction or neuropsychiatric disorders, and especially how drug abuse and other events during early life stages might lead to the formation of more vulnerable prefrontal network configurations. Finally, we show different promising pharmacological approaches that, when combined with the appropriate stimuli, will be able to re-establish these functional prefrontocortical configurations during adulthood.

## The mPFC as a High-Order Cognitive Area

A remaining question in the field of neuroscience is how our brain shapes the decision-making process, i.e., the ability to coordinate thought and action to achieve internal goals. Classic studies show the medial prefrontal cortex (mPFC) is an important region involved in the decision-making process, and patients with damage in the frontal lobe show deficits in attention and behavior control (Hagberg, 1987) and inability to acquire and use behavior-guiding rules (Shallice, 1982; Wise et al., 1996). Recent research, not only in humans but also in rodents, has shown that the mPFC is a key region for high executive functions such as rule learning and other different aspects related to working memory (Kesner and Churchwell, 2011). In this regard, lesion studies with damage in the prelimbic (PrL) and infralimbic (IL) cortices have been shown to produce deficits in both spatial (Horst and Laubach, 2009) and visual working memory (Di Pietro et al., 2004). Another task related to working memory is the temporal order memory; it requires the animal to remember the last visited arm in a maze and has also been shown to be dependent on the PrL and the anterior cingulate (AC) cortices (Chiba et al., 1997; Barker et al., 2007). Interestingly, the PrL cortex has also been suggested to mediate behavioral flexibility, i.e., the ability to change learned rules to obtain a reward, as in rotating rewards and inverting patterns for reward retrieval (Dias and Aggleton, 2000; Rich and Shapiro, 2007).

Regarding the decision-making process, lesions in the AC lead to disruptions in effort-based tasks, such as reduced willingness to climb a barrier to get a large reward (Rudebeck et al., 2006). Supporting these findings, effort-based decisions increased activity not only in the AC but also in the PrL and other prefrontocortical regions such as the lateral orbital cortex (Endepols et al., 2010). Similarly, the willingness to wait for a larger reward is also dependent on regions of the mPFC (Mobini et al., 2002; Churchwell et al., 2009).

## Top-Down Control of mPFC on Neuromodulation

One of the most accepted theories of how the mPFC controls the decision-making process is the top-down control from the mPFC (Miller and Cohen, 2001), which states that the mPFC provides biased signals representing the goals and the means to achieve those goals. These signals would be sent to efferent regions throughout the brain, including sensory or motor executive regions, but also those areas related to memory retrieval and emotions. These biased signals would determine how the flow of neural activity is guided to achieve those goals (Miller and Cohen, 2001). However, the mPFC also connects, both directly and indirectly, to important regions responsible for the modulation of mood, decision making and addiction, such as the ventral tegmental area (VTA) and the dorsal raphe nucleus (DRN; Russo and Nestler, 2013). This neuromodulatory route represents an alternative top-down pathway through which the mPFC controls behavior (Challis and Berton, 2015).

After the establishment of the physiological and computational principles underlying reward prediction coding (Schultz et al., 1997), a vast number of studies have reported that multiple behaviors function according to the reward prediction principles and its associated neuromodulatory circuits (Dayan et al., 2000). These networks are responsible for the learning of expected behavioral outcomes through the enhanced release of dopamine (DA) on specific locations (Schultz, 2016). While previously the focus has been set on the different routes through which DA is secreted: mesolimbic, mesocortical, nigrostriatal and tuberoinfundibular pathways (Luo and Huang, 2016), we want to stress the relevance of a reverse circuit in the regulation of certain behaviors related to addiction and neuropsychiatric disorders: from the mPFC to the striatum and then to the dopaminergic regions. In this line, the main efferent projections from the PrL target the caudate-putamen and the core of nucleus accumbens (NAcc), while the IL targets the medial shell of NAcc (Heidbreder and Groenewegen, 2003; Hoover and Vertes, 2007). Interestingly, the NAcc core and shell display different activities during the establishment of rewards: While the core is active before a newly-devalued cue, the shell decreases its activity (West and Carelli, 2016); hence granting both PrL and IL cortices different roles in the reward circuitry.

These basal ganglia have been long suggested to loop with the mPFC, being these loops key for the incentive learning and control of motor behaviors through activity in the substantia nigra (SN) and the VTA (Allen and Tsukahara, 1974; Lanciego et al., 2012; Leisman et al., 2014). These two regions are the source of dopaminergic transmission in the brain and have been suggested to feed back to the basal ganglia (Haber et al., 2000), pointing to the relevance of the mPFC in the DA circuitry. Therefore, the mPFC would exert an integrative role in processing motor and sensory inputs; and together with the basal ganglia, would be controlling the learning of appropriate and incentivized series of motor behaviors that include motor skills and habits (McNab and Klingberg, 2008; Graybiel and Grafton, 2015). Similarly, we suggest that a similar mechanism might exist, by which the integrative role of the mPFC would control higher cognitive behaviors as well.

In this line, the study of inputs to the VTA and the SN indicates strong projections from different areas such as the NAcc as indicated, but also from the dorsal striatum and the lateral hypothalamus (LH), among others (Geisler and Zahm, 2005; Watabe-Uchida et al., 2012). Interestingly, a similar pattern has been described when studying the input projections to the dorsal raphe, the area responsible for serotonergic innervation. Serotonin (5HT) has also been shown to modulate decision making, as well as to interact with the reward prediction error circuitry. Moreover, the altered expression of 5HT is associated with the onset of different mood disorders, including major depressive disorder (Challis and Berton, 2015). Interestingly, the areas projecting to the dorsal raphe include the ventral and dorsal striatum, the LH and the septum (Gabbott et al., 2005; Commons, 2016; Ogawa and Watabe-Uchida, 2018).

**Figure 1:**
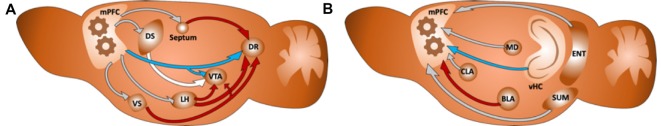
**(A)** Diagram of a sagittal section of the rodent brain illustrating how the integration of information in the medial prefrontal cortex (mPFC) affects both directly (in blue) and indirectly (in red) monoaminergic nuclei in the brain, as an example for the top-down control of the mPFC. The rest of the connections are colored in gray. **(B)** Diagram illustrating some of the main inputs to the mPFC. We have colored in red the projection from the BLA and in blue the projection from the vHC: two regions we hypothesize to compete for target innervation in the mPFC. The rest of the connections are colored in gray.

It is important to note that different parts of these dopaminergic and serotoninergic regions, as well as their specific inputs, have been suggested to mediate different functions of the prediction reward system (Ogawa and Watabe-Uchida, 2018). However, it is also relevant to support our hypothesis that most of the areas projecting to the dopaminergic and serotoninergic regions are also the main targets of the mPFC (Gabbott et al., 2005; see Figure 1).

The DRN is the main source of 5HT in the brain and receives dense monosynaptic input from the mPFC that contacts both serotoninergic and GABAergic neurons. Optogenetic activation of the mPFC onto the DRN produces strong disynaptic inhibition (Zhou et al., 2017). Nevertheless, the net effect of mPFC input on DRN activity, caused by the balance between direct excitation to serotonergic neurons vs. disynaptic inhibition, can be modulated not only by endocannabinoids (Geddes et al., 2016) but also by stressful experiences such as social defeat (Challis et al., 2014). A disrupted balance between excitation and inhibition (E/I) in the DRN after mPFC activation has been hypothesized to be an underlying factor for developing depressive-like symptomatology and that high-frequency deep brain stimulation (DBS) protocols of the mPFC (as performed in humans and rodents) can restore the E/I balance towards a higher excitation onto serotonergic neurons, thus enhancing DRN output and providing a mechanistic explanation for the antidepressant action of DBS of the mPFC (Challis and Berton, 2015).

Altogether, these data suggest that top-down control from the mPFC is exerted through the action of monoamines, such as DA) and 5HT. These two systems have been long suspected to play opposite roles in the reward circuitry (Solomon and Corbit, 1974), with DA reinforcing positive reward prediction error, and 5HT facilitating learning of new adaptive behaviors and inhibiting non-adaptive responses (Boureau and Dayan, 2011; Cools et al., 2011). Recent findings have shown that these processes are not only caused by the different effects of these neurotransmitters—5HT promoting LTD (He et al., 2015) and DA facilitating LTP (Li et al., 2003; Otani et al., 2003)—but are caused by their different release dynamics. In fact, in a conditioning paradigm, the action of DA is faster after reversal learning than that of 5HT. However, in that same experiment, DA was also shown to be withdrawn more rapidly during the negative prediction error, favoring the slow 5HT signals (Matias et al., 2017). Suggesting that DA reinforces a positive reward, but 5HT indicates a mismatch between expectations and reality.

Nevertheless, the mPFC is also interconnected to other regions that mediate the activity of neuromodulatory areas, such as the medial thalamus, or the periaqueductal gray area (Cameron et al., 1995; Vertes et al., 2012). In this line, a recent functional imaging study has shown that the connectivity between the mPFC and these regions is altered in the rumination of patients with chronic pain (Kucyi et al., 2014).

It is also important to note that an increasing amount of evidence is highlighting the relevance of critical periods for the establishment of functional connectivity between the mPFC and its afferent regions, and how such connectivity is disrupted in different psychiatric disorders such as addiction or depression (Crews et al., 2007; Contreras-Rodríguez et al., 2016; Pujol et al., 2019).

## Inputs to the mPFC

The data discussed here supports the classical view of the mPFC as a key player in our decision-making process. However, to better understand how it achieves these goals, we focus here not only on its outputs but also on its inputs. It has been long suggested that mPFC has a role in integrating diverse information from many different brain regions (Fuster, 1985, 1995). In this line, several studies have described the connectivity of the mPFC (Gabbott et al., 2005; Hoover and Vertes, 2007) revealing that the mPFC is a very interconnected structure within its different parts and with other regions, forming part of a brain-wide network of interconnected structures involved in mood control (Gilbert et al., 2010; Riga et al., 2014). It receives direct input from the ascending neuromodulatory monoaminergic systems, like the DRN, the VTA or the locus coeruleus. Also, its most dorsal part receives strong projections from the somatosensory and motor cortices, resulting in the motor response, or attention control (Passetti et al., 2002). On the other hand, the ventral parts are usually associated with cognitive spatial and mnemonic processes (Heidbreder and Groenewegen, 2003) receive strong projections from limbic structures, such as the hippocampal formation and the amygdala, as well as other regions such as the claustrum, the entorhinal cortex, the mediodorsal nucleus of the thalamus or the supramammillary nucleus in the hypothalamus (see Figure 1; Hoover and Vertes, 2007).

From these regions, the ventral Hippocampus (vHC) and the basolateral amygdala (BLA) are two of the most important regions conveying different types of information to the mPFC due to their role in different functions such as memory formation, spatial navigation or fear processing (Spellman and Gordon, 2015). We hypothesize that the integration that takes place during early life stages in the mPFC between these two different sources of information is a key factor, that determines the behavioral outcome of the animal in a given scenario, i.e., depending on the strength of each input and how it is processed by the mPFC.

## A Distributed Brain Network for Mood Control in Health and Disease

Focusing on these two regions directly projecting to the mPFC, the vHC has been traditionally thought to be important for emotional memories (Fanselow and Dong, 2010), although recent studies have shown that this area also manages spatial information (Kjelstrup et al., 2008; Wirt and Hyman, 2017). On the other hand, the BLA would convey more emotional-related information to be processed into the mPFC (Garcia et al., 1999; Senn et al., 2014), and is necessary to express fear-related behaviors such as freezing (Helmstetter and Bellgowan, 1994). Alterations of its connectivity in humans has already been suggested to underlie aggressive behaviors (Leutgeb et al., 2016).

Interestingly, when reviewing the literature, opposite alterations in these two structures can be found after different experimental conditions: patients suffering from major depression show reduced activity and volume in the hippocampus (Sheline et al., 1999; Campbell et al., 2004; Videbech and Ravnkilde, 2004; Milne et al., 2012), but an increase in these parameters when measured in the amygdala (Anand et al., 2005; Hamilton et al., 2012). In animal models of depression, such as chronic or prenatal stress, animals show dendritic atrophy in the hippocampus (Sousa et al., 2000; Mychasiuk et al., 2012) and dendritic growth in the amygdala (Vyas et al., 2002). These changes can be reversed by antidepressants such as Fluoxetine (Magariños et al., 1999; McEwen and Chattarji, 2004), by promoting an increase in spine density of pyramidal neurons in the hippocampus (Hajszan et al., 2005). Fluoxetine and other antidepressants also produce an increase in BDNF mRNA in the hippocampus (Nibuya et al., 1995; Larsen et al., 2008). While stress produces a decrease in BDNF in the hippocampus, it leads to an increase in the amygdala (Lakshminarasimhan and Chattarji, 2012), suggesting an important role of BDNF in the structural changes observed.

In humans, a common polymorphism in the BDNF gene is the substitution of Val to Met at codon 66, known as Val66Met (Shimizu et al., 2004). This allele has been associated with several neuropsychiatric disorders (Harrisberger et al., 2015). Interestingly it has been shown that this polymorphism of BDNF is associated with an increased activity of the amygdala and, conversely, a decreased activity in the hippocampus. This has been suggested as the underlying reason for an impaired fear extinction in patients and slightly impaired memory retrieval (Hariri et al., 2003; Soliman et al., 2010; Hajek et al., 2012).

Furthermore, recent evidence measuring functional connectivity after fear acquisition shows that the connectivity between the amygdala and the mPFC is decreased during fear memory consolidation, while that between the hippocampus and the insular cortex, another important region in the decision-making process (Droutman et al., 2015; Von Siebenthal et al., 2017), is enhanced (Feng et al., 2014).

Together, these results point to a prefrontocortical network configuration in major depression dominated by a reduced functional connectivity with the amygdala. Interestingly, an increased functional connectivity between the prefrontal cortex and the amygdala have been reported in animal models of autism (Huang et al., 2016) as well as in human subjects with autism spectrum disorders (Iidaka et al., 2019). Highlighting the relevance of precise mechanisms controlling the functional connectivity balance across this distributed network of brain structures for mood control. Furthermore, prefrontocortical configurations associated with the disease not only span top-down afferents but also hyperconnectivity and hyperplasticity of local microcircuits have been reported in animal models of autism (Rinaldi et al., 2008).

On the other hand, recent evidence has shown that certain pharmacological treatments, such as antidepressants, seem to reverse certain pathological network configuration, through structural plasticity dependent mechanisms, to a balance of these two inputs and, interestingly, when administered to control animals, it leads to a hippocampal dominated network configuration. It has been shown recently that the activation of the projection from the vHC to the mPFC both optogenetically and chemogenetically in the adult brain, can replicate the effects of the fast-acting antidepressant ketamine for a short time (Carreno et al., 2016).

## A Critical Period of Opportunity

Critical periods are defined as temporal windows of enhanced plasticity in brain development, during which a specific circuit or region is highly sensitive to experience (Hensch, 2005).

It has been shown in several sensory cortices that, during these critical periods, the incoming developing inputs compete in an experience-dependent manner for target innervation: i.e., left and right eye compete for innervation of the visual cortex (Hubel and Wiesel, 1970) or different whiskers compete for space in the barrel cortex (Van der Loos and Woolsey, 1973).

During the critical period of the fear system, at a time point when fear memories can be extinct easily, there is an increase in the connectivity from the vHC to the mPFC (Pattwell et al., 2016), which is in agreement with other studies suggesting that the projection from the ventral hippocampus to the mPFC can disrupt expression of fear memories (Sotres-Bayon et al., 2012). We have shown evidence for the vHC and the BLA competing for target innervation in the mPFC (Guirado et al., 2016). In this line, we hypothesize that the mPFC has a critical period in which its different incoming inputs compete in an experience-dependent fashion. Moreover, the specific results of this competition would determine a specific prefrontal network configuration, which, we believe, is a key element to understand neurodevelopmental trajectories to different psychiatric disorders.

Interestingly, not only the inputs onto the mPFC undergo late development and susceptibility during this critical period. It has been shown that the prefrontal-amygdalar output undergoes late development in mice (Arruda-Carvalho et al., 2017) as late as 45 days of postnatal development. Moreover, the increase in serotonergic tone, produced by the blockage of serotonin transporter by antidepressants during this period, has been shown to increase the output innervation from the mPFC to the DRN (Soiza-Reilly et al., 2019). It is worth noticing that the alteration in the strength of mPFC projections to the amygdala and the DRN has been shown as a factor associated with increased risk to develop anxiety and depression (Chen et al., 2018; Soiza-Reilly et al., 2019).

In line with the idea of a critical period in the mPFC, both classical and fast-acting antidepressants, as well as optogenetic and chemogenetic stimulations, only revert temporarily the behavior in paradigms of mental diseases (Carreno et al., 2016). Therefore, we propose that to achieve long-lasting effects, a critical period plasticity context is required for the remodeling of prefrontal network configurations. Most animal models of neuropsychiatric disorders are based on manipulations during early life and/or adolescence (Nestler and Hyman, 2010; Andersen, 2015), probably during that critical period in the mPFC.

**Figure 2:**
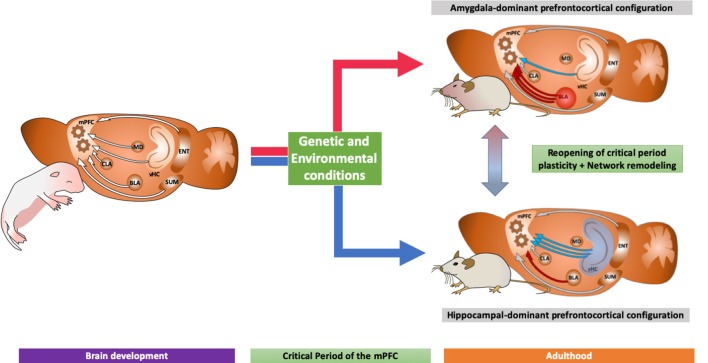
Diagram showing two different neurodevelopmental trajectories according to its prefrontocortical inputs: (1) an amygdaloid-dominant scenario, with strong projections from the basolateral amygdala to the mPFC, with increased volume in the amygdala which, as discussed in the main text, is related to increased fear expression and neuropsychiatric disorders; and (2) a hippocampal-dominated network, associated to antidepressant action and increased neuroplasticity. We also indicate the possibility of new treatments able to revert the network configuration as explained in the main text.

We hypothesize that after the closure of this critical period, the brain will remain with a specific network configuration according to the strength of its mPFC inputs during such a time window. Moreover, we suggest two specific mPFC configurations: one dominated by the amygdala and one by the ventral hippocampus (see Figure 2). These specific configurations can be correlated with specific behaviors through adulthood, as discussed above, building a bridge between behavior and network connectivity that could explain, to a certain extent, the decision-making process.

Epidemiological data have shown that early life trauma, including physical abuse, sexual abuse or neglectful parenting is correlated not only with alcohol abuse (Clark et al., 1997), but also to other substances, including THC (Bensley et al., 1999; Dube et al., 2003). Moreover, vulnerability to stress and depression has been associated with rodents with increased drug abuse (Riga et al., 2018), showing the interconnectivity of these disorders and a possible common origin. In this line, we propose here that aversive experiences during early life lead to a prefrontocortical network dominated by the amygdala, which would be more vulnerable to brain diseases such as neuropsychiatric disorders or addiction. The impact of early life trauma seems to be restricted to certain time windows, supporting the idea of a prefrontal cortex critical period (Rutter et al., 2007).

The good news is that the combination of drugs that reopen critical period plasticity, such as Fluoxetine (Guirado and Castrén, 2018), with the proper experimental conditions, can effectively allow the network configuration to change to a healthy state during adulthood. We have proven this principle both in the visual (Maya Vetencourt et al., 2008) and in the fear systems (Karpova et al., 2011).

Moreover, we have recently found that the combination of behavioral therapy (re-socialization in isolated animals) after chronic Fluoxetine treatment, increases the strength of the projection from the vHC to the mPFC and reduces abnormal aggressive behavior in a long-time manner (Mikics et al., 2018). Interestingly, other substances whose efficiency as antidepressants is undergoing a revision both in the context of clinical treatment as well as basic research, such as MDMA (Nardou et al., 2019), ketamine (Berman et al., 2000; Li et al., 2010) or isoflurane (Antila et al., 2017), have been shown to reopen a critical period for social behavior through different molecular mechanisms of plasticity, including oxytocin, mTOR and TrkB (Li et al., 2010; Antila et al., 2017; Nardou et al., 2019).

Thus, new approaches using drugs aiming at the reopening of critical periods of plasticity (Castrén and Antila, 2017; Guirado and Castrén, 2018), together with environmental conditions or the experimental manipulation of the activity of these prefrontal inputs, will provide a new proof of concept to explore new and efficient treatments for addiction and neuropsychiatric disorders. Furthermore, future research studying how different inputs are integrated and processed in the mPFC will help us understand further the decision making process and the human mind.

## Author Contributions

RG has written the draft and designed the review. All authors have contributed significantly reviewing the manuscript.

## Conflict of Interest

The authors declare that the research was conducted in the absence of any commercial or financial relationships that could be construed as a potential conflict of interest.

The handling editor is currently co-organizing a Research Topic with one of the authors AF, and confirms the absence of any other collaboration.
